# Complement, thrombotic microangiopathy and disseminated intravascular coagulation

**DOI:** 10.1186/s40560-014-0061-4

**Published:** 2014-12-31

**Authors:** Shinichiro Kurosawa, Deborah J Stearns-Kurosawa

**Affiliations:** Boston University School of Medicine, 670 Albany Street, Boston, MA 02118 USA

**Keywords:** Complement, Blood coagulation, Thrombotic microangiopathy, Disseminated intravascular coagulation, Hemolytic uremic syndrome, Thrombocytopenia, Microangiopathic hemolytic anemia, Acute kidney injury

## Abstract

In the blurring boundaries between clinical practice and scientific observations, it is increasingly attractive to propose shared disease mechanisms that could explain clinical experience. With the advent of available therapeutic options for complement inhibition, there is a push for more widespread application in patients, despite a lack of clinically relevant research. Patients with disseminated intravascular coagulation (DIC) and thrombotic microangiopathies (TMA) frequently exhibit complement activation and share the clinical consequences of thrombocytopenia, microangiopathic hemolytic anemia, and microvascular thrombosis. However, they arise from very different molecular etiologies giving rise to cautious questions about inclusive treatment approaches because most clinical observations are associative and not cause-and-effect. Complement inhibition is successful in many cases of atypical hemolytic uremic syndrome, greatly reducing morbidity and mortality of patients by minimizing thrombocytopenia, microangiopathic hemolytic anemia, and microvascular thrombosis. But is this success due to targeting disease etiology or because complement is a sufficiently systemic target or both? These questions are important because complement activation and similar clinical features also are observed in many DIC patients, and there are mounting calls for systemic inhibition of complement mediators despite the enormous differences in the primary diseases complicated by DIC. We are in great need of thoughtful and standardized assessment with respect to both beneficial and potentially harmful consequences of complement activation in these patient populations. In this review, we discuss about what needs to be done in terms of establishing the strategy for complement inhibition in TMA and DIC, based on the current knowledge.

## Introduction

The complement and coagulation systems are considered to be descended from a common ancestral system, more than 400 million years ago [1,2]. The common feature of these two cascade systems is that both systems are activated by common activators or host conditions [3]. Both systems contain a series of serine protease-mediated reactions, and there is evidence for networking crosstalk with shared activators and inhibitors. Members of each cascade interact either directly or indirectly. Upon bacterial infection, acute blood loss, trauma with tissue injury, malignancy, and many other underlining diseases, both the coagulation and complement systems are activated in patients and animal models. If these processes escape from their tight and localized control, it may lead to systemic inflammatory response syndrome (SIRS) and multiple organ failure, which is a major contributor to high mortality.

## Review

### Cross talk between complement and coagulation systems

**Figure 1:**
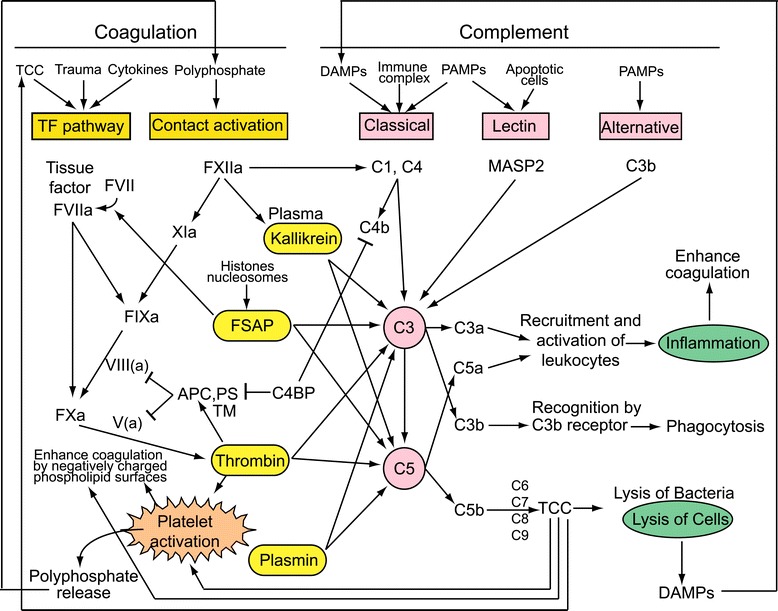
**Crosstalks between coagulation, fibrinolysis and complement systems.** The coagulation cascade is roughly divided into TF pathway and contact activation. The TF pathway is well known to get activated by TCC, trauma, and some cytokines. Both pathways will merge at FXa level, which will generate thrombin. Thrombin is one of the most potent activator of platelets. Upon platelet activation, medium-size polyphosphate in the platelet granules will be released, which can induce contact activation. FXIIa can activate the classical complement pathway. FXIIa can activate plasma kallikrein, which in turn can activate both C3 and C5. Other members of blood coagulation and fibrinolysis, such as FSAP, thrombin and plasmin can independently activate both C3 and C5. DAMPs, immune complex and PAMPs are known to activate the classical complement pathway. PAMPs and apoptotic cells will activate lectin pathway. PAMPs will trigger alternative pathway activation, all leading to C3 activation, which will activate C5. C3a and C5a will recruit and activate leukocytes, as well as induce platelet activation and aggregation, inducing thrombosis and inflammation, which are known to further enhance coagulation. C5b will lead to TCC formation, which not only lyse microorganisms but also lyse host cells, which will release DAMPs. TCC will induce TF pathway, induce platelet activation, and enhance coagulation by negatively charged phospholipid surfaces.

The interplay between the two systems has been studied for decades [4-7], and this review will highlight the more current literature (Figure 1). Multiple components of the complement cascade have the capacity to alter the phospholipid composition of the outer membranes of cells. The terminal complement complex (TCC or C5b-9 complex) can flip phosphatidylserine from the internal leaflet to the cell exterior surface, thereby providing a negatively charged surface necessary to support the coagulation cascade. Complement C3a induces platelet activation and aggregation [8]. Before activation, the outer leaflets of cell and platelet membranes normally do not contain negatively charged phospholipids, whereas abundant phosphatidylserine becomes available after activation and these surfaces support coagulation. Cell activation will also release granular contents, which generally enhance pro-coagulant responses, and release of microparticles will provide extra surfaces for clot formation.

Binding of C1q to platelets induces the expression of integrins and P-selectin [9]. The platelet and C1q interaction seemed to be more complicated than just moderate and transient upregulation of P-selectin. Pre-incubation of platelets with C1q, on the other hand, will diminish collagen-induced upregulation of P-selectin, but the pre-incubation is reported to potentiate the collagen-provoked production of reactive oxygen species. This paradoxical C1q modulation of platelet that is observed *in vitro* may play a significant role in the pathogenesis of many complement diseases, given that the role of platelet not only resides in hemostasis but platelet also interact with white blood cells and modulate immune and inflammatory reactions.

On endothelial cells, complement effectors will change the cell properties from anti-coagulant to pro-coagulant. Complement C5a can induce/increase tissue factor expression in various cell types, including endothelial cells [10], and TCC or C1q will upregulate several adhesion molecules.

Thrombin, the end-product of the coagulation cascade, has considerable capacity to accelerate activation of both pathways by forming positive feedback loops. Thrombin generates C5a, a powerful anaphylatoxin, in C3 null mice lacking C3 convertase [11], and C5a induces tissue factor activity on human umbilical vein endothelial cells. Similarly, immunoblockade of C5 activation reduces microparticle-associated tissue factor activity and antigen as a consequence of *Neisseria meningitides*, the cause of bacterial meningitis [12]. Activation of complement was shown to decrypt encrypted tissue factor [13]. Activation of platelets will induce its granular release. One of the most abundant substances is polyphosphate. Platelet polyphosphate is much shorter than microbial long-chain polyphosphate, and platelet polyphosphate is demonstrated to exert contact activation of blood coagulation [14].

Kallikrein and factor XIIa can cleave complement components. Plasmin, the most powerful serine protease of the fibrinolytic system, was also shown to be capable of cleaving both C3 and C5, and the cleavage products are biologically active [15].

Factor VII-activating protease (FSAP), also known as plasma hyaluronan binding protein, is a circulating serine protease believed to activate blood coagulation factor VII and single-chain pro-urokinase [16], although activation of factor VII is questioned [17]. FSAP zymogen is notoriously unstable and can be activated by histones and nucleosomes arising from necrotic or apoptotic cells. Trauma patients have high levels of these circulating damage-associated molecular pattern (DAMPs) molecules, and FSAP is activated in patients with multiple traumas [18]. Complement proteins form complexes with FSAP and FSAP can cleave C3 and C5 to generate the C3a and C5a anaphylatoxins [15].

**Figure 2:**
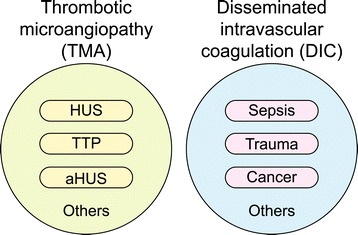
**Venn diagram of thrombotic microangiopathy and disseminated intravascular coagulation.** As described in the text, the authors used the term “TMA”, which excludes DIC. The most popular thrombotic microangiopathy is HUS, which involves Shiga toxin. Atypical HUS is caused by chronic, uncontrolled, and excessive activation of complement-inducing platelet activation, endothelial injury, white cell recruitment, and activation, leading to TMA. Most cases of TTP arise from inhibition of the enzyme ADAMTS13, a metalloprotease responsible for cleaving large multimers of von Willebrand factor. A rare form of TTP is caused by genetically inherited dysfunction of ADAMTS13. This form is called Upshaw-Schülman syndrome. DIC is not a distinct disease entity. It occurs as a secondary complication of many different disorders, including sepsis, trauma, cancer, obstetric complications, and others.

In addition to the direct molecular links between the coagulation and complement systems, both systems are intimately linked to inflammation. Activation of the complement and coagulation systems is capable of independently augmenting inflammatory responses, which in turn can potentiate both complement and coagulation activation. Endothelial cells play major roles in inflammation, including endothelial cell activation and injury/dysfunction. Given the abundant cross talk between coagulation and complement systems, it seems unlikely that one will be activated without the other. The expectation is that complement will be activated in almost all thrombotic disorders, including DIC and TMA, both of which can develop with thrombocytopenia, microangiopathic hemolytic anemia, and microvascular thrombosis. Thus, some groups of experts in the field place DIC in the list of TMA in a broader sense. However, in this article, DIC will be considered to be distinct from the TMAs and will be included in the list of differential diagnoses because most cases are distinguishable based on clinical presentation and laboratory findings (Figure 2). However, as occurs many times in clinical practice, these grouping designations still present a challenge in a small number of patients, especially in the presence of overlapping clinical features.

### Thrombotic microangiopathy

TMA involves several distinct etiologic pathological processes, but with shared clinical features that include thrombocytopenia, microangiopathic hemolytic anemia, and microvascular thrombosis, leading to end-organ ischemia, infarction, and dysfunction. The most common TMA is due to hemolytic uremic syndrome (HUS), caused by Shiga toxin (Stx)-producing enterohemorrhagic *Escherichia coli* (EHEC). Infection with this toxigenic intestinal pathogen is a leading cause of acute renal failure in otherwise healthy children. EHEC can produce two Shiga-like exotoxins, Stx1 and Stx2, which are primary virulence factors causing organ injury [19]. These are ribosome-inactivating toxins, named after Dr. Kiyoshi Shiga, a pioneering clinical microbiologist born in Sendai, who made the landmark discovery at the turn of the 20th century that “toxic factors” from *Shigella dysenteriae* cause the clinical manifestations of bacillary dysentery (*sekiri*) [20]. Shiga toxin from *S. dysenteriae* is the prototype of this family of toxins, and Stx1 from EHEC differs by only one amino acid. Stx2 shares 56% amino acid sequence identity with Stx1, and the toxins are antigenically distinct [21]. Many clinical and animal studies have shown that Stx is necessary and sufficient for causing HUS, and for reasons that are not well understood, Stx2 is associated with more severe clinical consequences. Other, more rare, causes of TMA-associated HUS include invasive pneumococcal infection in pediatric patients [22,23], atypical HUS, thrombotic thrombocytopenic purpura (TTP), and others.

### HUS

HUS arising during EHEC infection is a global public health problem. The massive EHEC outbreak due to contaminated school lunches in over 12,000 symptomatic adults and children in Sakai City, Osaka, Japan, in 1996 is one of the largest outbreaks known [24]. During the spring and summer of 2011, there was an outbreak of hemorrhagic colitis and HUS in Europe, originating in Germany, involving more than 4,000 cases, 852 patients with HUS and 54 deaths. It was caused by bean sprouts contaminated with an enteroaggregative *E. coli* strain that had acquired the ability to produce Stx2 [25]. Argentina has arguably the highest incidence of EHEC infection and pediatric HUS [26,27] and contaminated ground beef, water, fresh produce, and other foods that causes recurrent global public health problems. According to the US Centers for Disease Control and Prevention, the human burden is approximately 110,000 infections annually in the United States alone [28] and is estimated at approximately 1.5 million cases globally with 3%–5% mortality and significant morbidity, including impaired renal function, hypertension and cardiovascular diseases, pre-eclampsia, and other complications.

Clinically, HUS from any etiology includes a thrombotic component and is considered to be a TMA and distinct from DIC. However, DIC and TMA share extensive features, including thrombocytopenia, microvascular thrombosis, microangiopathic hemolytic anemia, and organ dysfunction, such as acute kidney injury. This is why some groups prefer to include DIC in the list of TMAs. In order to dissect the differences and similarities between DIC and TMA, the simplest approach is to examine animal models of TMA and DIC. Although there are multiple DIC animal models, animal models of TMA have significant challenges when the goal is to recapitulate human responses.

Injection of Stx into mice results in acute kidney injury but, unlike humans, the Stx challenge does not cause thrombocytopenia or TMA. This is thought to be due to the fact that murine glomerular endothelial cells do not express globotriaosylceramide (Gb3) glycolipid, the receptor for Shiga toxin [29]. As a result, glomerulopathy is not the main pathology in mice Stx model. Instead, mouse renal tubular cells express the receptor, leading to direct tubular lesions and kidney dysfunction. The mouse Stx model is a very good model for screening compounds that will neutralize Stx, for example. However, the mouse Stx model is not a good model for studying TMA because Stx does not cause TMA in mice. In order to circumvent this shortcoming, a murine Stx + LPS model was proposed, in which LPS is co-administered together with Stx [30]. This model does show complement activation, thrombocytopenia, and glomerulopathy, which are missing in the mouse Stx model, and there has been inferences that LPS must be a critical component in humans because it is true in mice. Unfortunately, most researchers in the field of EHEC-HUS have limited clinical or research experience with DIC. It has been well established for decades that LPS activates complement and causes DIC in animals and humans [31] and is clinically distinct from HUS.

Complement is activated in the LPS + Stx murine model and that coupled with evidence of complement in some EHEC patients [32] further suggested that complement must be driving the TMA in HUS.

### Atypical HUS

Atypical HUS is a very rare progressive and life-threatening disease. Most patients have genetic abnormalities in the complement system, with most of the mutations found in the regulatory molecules of complement activation. It is thought that chronic, uncontrolled, and excessive activation of complement causes platelet activation, endothelial injury, white cell recruitment, and activation, all of which lead to TMA.

The role of complement activation in atypical HUS is very well established. The end results produce essentially the same clinical features, including consumptive thrombocytopenia, microangiopathic hemolytic anemia, microvascular thrombosis, and TMA, with the exception of the time course. There is a treatment available, and it is very effective. The drug is called Eculizumab™, a humanized monoclonal antibody to complement 5 (C5). By binding to C5 and inhibiting its activation, the antibody ultimately prevents terminal complement complex (TCC) formation, providing the needed regulation of the complement pathway.

### Is Stx-HUS driven by complement activation?

Since both HUS and aHUS present with reasonably similar clinical parameters, except for time course, in combination with the observation that complement is activated in the murine Stx + LPS co-injection model, there is thinking that complement activation is the common driver of coagulopathy in HUS, atypical HUS, and DIC [32]. This approach is intended to explain why HUS and atypical HUS have overlapping clinical presentations with consumptive thrombocytopenia, microangiopathic hemolytic anemia, microvascular thrombosis, and TMA. Moreover, some patients with EHEC infection and HUS show evidence of complement activation. However, while there is no doubt that complement regulation is the villain in atypical HUS, the actual evidence for complement as a primary mediator of HUS or TTP is weak.

Eculizumab™ was approved for off-label use on a compassionate basis for use in a sub-group of patients with particularly refractory HUS during the 2011 EHEC outbreak in Europe (University Hospital of Bordeaux Regional Ethics Committee, and AFSSAPS). The results were mixed. While it is clear that the drug can be safely used in patients with HUS, it was not clear whether the treatment was effective or not [33]. This was a study done under remarkable conditions, performed in the middle of a public health emergency in several countries, so control groups were not possible and efficacy was difficult to conclude.

The question of complement involvement in HUS continues in part because there is no specific treatment available, other than general intensive supportive care, including dialysis and administration of intravenous fluids [34]. If complement activation plays a major role in HUS, then Eculizumab™ has the potential to become the first drug for the treatment of HUS associated with EHEC. Since EHEC-HUS is the most frequent among TMAs, specific treatment will be well received by patients, families, and physicians. Proof of efficacy would require randomized double-blind placebo controlled clinical trials which will be very expensive and need a substantial commitment, given that most patients will be children and will present with acute and emergency courses. Even though the incidence of EHEC infection is estimated to be about 110,000 annually in the US, or 1.5 million globally, these are usually small outbreaks and the expected number of patients per one health care facility is typically small. A clinical trial will require many centers and a long period of time, further adding more costs. To make the matters even worse, HUS occurs in only about approximately 8% of EHEC cases, increasing the necessary enrollment number exponentially to the point that investors will be reluctant due to the limited market share and a limited return on a necessary substantial investment.

### Non-human primate model of Stx-HUS

To help relieve this bottleneck, our laboratory asked the question of whether complement activation is required for the development of HUS. We used our non-human primate (NHP) model of Stx-induced HUS. The baboons receive a single intravenous bolus injection of Stx1 or Stx2, and development of pathophysiology is monitored over time [35]. The initial dose response study revealed that Stx1 at 10 ng/kg elicited mild transient changes; 50 ng/kg was severe (4/5 euthanized), and 100 ng/kg was lethal (5/5 euthanized). The dose-response for Stx2 could also be titrated, but the concentration range differed with a lethal dose at 50 ng/kg Stx2 (6/6 euthanized). Subsequent studies revealed that there are significant differences between the toxins in timing, inflammation responses, and kidney pathology, but they share the ability to induce HUS [36-38].

Thrombocytopenia is one of the hallmarks of HUS, monitored closely in patients, and we also find platelet levels to be a good marker of disease onset and severity in the baboon models. In our model, both Stx1 and Stx 2 induced thrombocytopenia in a dose-dependent manner in all subjects. The levels of blood urea nitrogen (BUN) also increased dose dependently. Microscopic examination of the kidneys revealed microthrombi in the glomerular capillary, mostly made up of platelets or platelets and RBC [36]. Glomerular endothelial cells were lost or markedly swollen, with frequent fibrin deposition on the luminal side of basement membrane in the renal glomerular capillaries. D-dimer levels increase indicating that both coagulation and fibrinolysis takes place. In plasma and urine, we measured two damage-associated molecular patterns markers of cell injury, HMGB1 and histones, and found that both are elevated in our model of Stx-HUS, yet complement was not activated [38]. We measured plasma TCC levels and found no increase despite development of HUS and renal injury, indicating that there is no substantial complement activation throughout the entire course of developing HUS pathophysiology. This demonstrates that complement activation is not required for the development of HUS. The lack of complement activation in the *Citrobacter rodentium*-Stx2 murine model, which has an intestinal bacteria secreting Stx2 [39], is supportive of this notion, but again, the mice do not develop TMA. The baboon studies are limited by the fact that the baboons receive a single Stx challenge, rather than prolonged exposure to toxin from an enteric bacterial infection, and humans might just simply differ in response.

### Complement activation in DIC

In contrast to the baboon HUS model, plasma TCC levels elevate rapidly after intravenous challenge of baboons with Gram-negative or Gram-positive bacteria [38]. These bacteremia sepsis models are also consistent and reproducible models of DIC [40]. D-dimer increases, fibrinogen is consumed, and clotting times prolong. Similar to bacteremia, multiple trauma injuries are known to induce rapid activation of complement in humans [41,42]. Since we know that uncontrolled activation of complement can induce atypical HUS, and that there is substantial complement activation in DIC models [43] as well as patients, it is conceivable that DIC patients may suffer the same pathology as that observed in patients with atypical HUS, in addition to DIC-specific pathology. Also, the extent of complement involvement may differ between each DIC patient. Since atypical HUS involves thrombocytopenia, microangiopathic hemolytic anemia, and acute kidney injury, there is a real possibility that Eculizmab™ may provide at least partial relief in certain sub-groups of DIC patients, including maybe burn injury patients [44].

It has been well over 100 years since complement was discovered. Complement activation in patients with DIC is well known, as is the crosstalk between the complement and coagulation systems. Given this networking, complement is activated in most patients with DIC, in which coagulation is most always activated. Due to the fact that complement is an immutable part of innate immunity involved in pathogen sensing, opsonization, and clearance, patients with infections may not be a good target population for a complement inhibiting strategy because we do not want to compromise innate immune response while the patients are trying to fight off the infections. However, the literature is conflicting, and whether C5b-9 is protective or detrimental remains to be determined [45]. Complement activation seemed less pronounced in isolated models of ischemia and reperfusion, whereas the responses are stronger in hemorrhagic trauma shock and multiple trauma injury models. The combination of traumatic brain injury and shock results in an immediate activation of coagulation and complement systems [46]. In humans, a number of reports indicate that the pathogenesis of spinal cord injury involves not only by primary mechanical trauma but also by secondary response, including complement activation [47]. Complement activation has been associated with acute neuroinflammation and secondary brain injury after severe trauma [48]. Mice lacking the receptors CR2/CD21 and CR1/CD35 are protected from adverse sequelae of experimental closed head injury [49].

### Complement inhibitors, current and future

Eculizmab™ is already in the market and several clinical trials for a variety of complement-driven diseases are ongoing [50]. Anti-C5a was shown to ameliorate coagulation and fibrinolytic changes in a rat model of sepsis [51]. C1 inhibitor may have a beneficial effect in a primate sepsis model [52]. Several other complement inhibitors are under development. TT30 is a novel therapeutic fusion protein linking the C3 fragment-binding domain of human complement receptor type 2 (CR2/CD21) with the complement alternative pathway inhibitory domain of human factor H [53]. TT30 is designed to deliver cell surface-targeted regulation of the alternative pathway activity. It blocks *ex vivo* hemolysis of paroxysmal nocturnal hemoglobinuria erythrocytes, while at the same time retaining the normal ability of the complement system to efficiently activate C3 through the classical and lectin pathways. The murine analog of TT30 was able to attenuate collagen-induced arthritis, systemic lupus erythematosus, and tissue injury [54]. Finally, anti-C3b/iC3b monoclonal antibody 3E7 can block both complement-mediated hemolysis and C3 deposition in an *in vitro* model of alternative pathway-mediated hemolysis [55].

The urgent question is: can complement activation in DIC be a target for treatment or not? Given that Eculizmab™ is already in the market, and many other complement inhibitors are in the short pipeline, the question needs to be answered rather quickly. We would like to come to the correct answer, preferably with ample evidences using pre-clinical DIC models, before application to humans. The problem is that we do not have ample evidences available at this point. It is highly unlikely that it will be a simple yes/no answer that will apply to all DIC patients. Our prediction is that it probably depends on cases, timing, and everything else. So the question will most likely become “which subcategory of patients with DIC will benefit, and which will not?” These types of questions will demand an enormous amount of data to make successful translation.

## Conclusions

Complement activation is observed in certain groups of TMA patients, as well as many patients with DIC. As the therapeutic options are expanding, more questions arise as to which patients may benefit from complement-targeted treatments. Since the complement pathway constitutes one of the most important innate immune effectors, it becomes critical to assess the role and the extent of complement activation in each patient. In order to get there, we need to study the extent of complement involvement in HUS, DIC, and TMA in pre-clinical animal models, as well as developing diagnostic methods for the assessment of patients.

## Abbreviations

DIC: disseminated intravascular coagulation
TMA: thrombotic microangiopathy
SIRS: systemic inflammatory response syndrome
TCC: terminal complement complex
C1q: complement component 1 q subcomponent
C3: complement component 3
C5: complement component 5
FSAP: factor seven activating protease
Stx: Shiga toxin
EHEC: enterohemorrhagic *Escherichia coli*
HUS: hemolytic uremic syndrome
p-HUS: HUS associated with invasive pneumococcal disease
TTP: thrombotic thrombocytopenic purpura
TF: tissue factor
Gb3: globotriaosylceramide (CD77)
BUN: blood urea nitrogen
DAMPs: damage-associated molecular patterns
CR2/CD21: complement receptor type 2
